# The influence of moral identity on green consumption

**DOI:** 10.3389/fpsyg.2022.1020333

**Published:** 2022-10-05

**Authors:** Dalin Li, Guo Cheng, Chunya Wang

**Affiliations:** ^1^Center for Energy Economic Research, School of Business Administration, Henan Polytechnic University, Jiaozuo, China; ^2^Business School, Sichuan University, Chengdu, China

**Keywords:** moral identity, advertising goal framing, moral awareness, self-consistency, social approval, green purchasing intention

## Abstract

Existing studies show that moral identity promotes green consumption, but its influence mechanism and boundary conditions remain unidentified. However, moral identity includes internalization and symbolization, which affect green consumption in different ways. Advertising goal framing also has an impact on green consumption. Existing studies often focus on moral identity internalization but neglect the role of moral identity symbolization and fail to fully consider the interaction, mechanism, and boundary conditions between moral identity and advertising goal framing. This study assumes that advertising goal framing includes both intrinsic and extrinsic goals. We attempt to verify the combined influence of internalization and symbolization of moral identity, advertising goal framing, and moral awareness on green purchasing intention. Through two experiments, we found that: (1) the internalization and symbolization of moral identity can promote consumers’ green purchasing intention; (2) consumers with high internalization of moral identity have more positive responses to intrinsic goal advertising; (3) moral awareness can enhance the combined influence of moral identity internalization and intrinsic goal advertising on consumers’ green purchasing intention; and (4) self-consistency and social approval mediate the combined impact of moral identity and advertising goal framing on consumers’ green purchasing intention. This study enriches the literature on the influence of moral identity on green consumption. Businesses can also draw on these findings to improve the effectiveness of green marketing.

## Introduction

Human consumption has a substantial impact on the environment. Changes in human production and lifestyle can effectively reduce carbon emissions and address climate change. The Chinese government has increasingly recognized the importance of the development of climate safety and environmental friendliness. The government attaches great importance to the institutionalization of “green” lifestyles for consumers, including efforts to advance green product purchasing. Green products conserve natural resources, use fewer toxic substances, emit fewer pollutants, and generate less waste during manufacturing (Amatulli et al., 2019). In response to policymaking, companies have launched a variety of green products, such as “slow fashion” clothing, energy-efficient home appliances, and organic food, to enhance their brand image and take advantage of market opportunities. However, green products are frequently more expensive than conventional products or lacking in performance and quality; therefore, consumers frequently face a conflict between self-interest and public interest when purchasing green products (Chuang et al., 2016), resulting in low market awareness and penetration. Hence, learning more about the processes that lead to green consumption is necessary.

Moral identity can promote consumers’ green purchasing intentions (Wu and Yang, 2018). Certain dimensions of moral identity, including internalization and symbolization, have a critical impact on green purchasing intention (Legere and Kang, 2020). For example, advertising that appeals to self-interest seems to have a greater influence on green purchasing intentions in a private consumption context. In contrast, altruistic appeals are more influential in the context of increased public responsibility (Green and Peloza, 2014). Abstract appeals within green advertising are more persuasive when associated with the interests of others (Yang et al., 2015).

As opposed to established variables such as “intention,” “subjective norm,” “perceived behavioral control,” and “past behavior,” the autonomous motivation, as outlined in self-determination theory (SDT), may be a more accurate predictor of consumer behavior (Webb et al., 2013). This is because theories describing consumer behavior prior to SDT overemphasize extrinsic goals and underemphasize intrinsic motivation (Webb et al., 2013). There is a framing effect between intrinsic and extrinsic goals in social marketing advertising (Seungae and Pounders, 2018). However, the following theoretical gaps in existing research still need to be addressed. First, the existing literature on moral identity has focused more on the effects of moral identity internalization and has not paid sufficient attention to moral identity symbolization. Therefore, it is unclear whether moral identity internalization and symbolization have the same effect on green consumption. Second, the existing literature often fails to consider the impact of green advertising when studying the impact of moral identity on green consumption. This is not in line with the real-world context of green consumption; thus, studies that assess a combination of personality traits and external stimuli are urgently needed. Third, the existing framework of green consumption goal framing is divided into self-interest and altruism goals, as well as gain, hedonic, and normative goals. There is a need for a new definition of advertising goal framing based on novel theories and to study the combined impact of advertising goal framing and moral identity on green consumption. Fourth, the existing literature lacks research on the boundary conditions and mediating mechanisms of the combined role of moral identity and advertising goal framing.

While both moral identity internalization and symbolization promote green consumption, their mechanisms of action are distinct. Moral identity internalization motivates consumers to strive for green consumption due to their own subjective moral inclinations. Consumers with a high moral identity symbolization are more persuaded by impression management to purchase green products. For goal framing within advertising, consumers with high moral identity internalization are more motivated to achieve intrinsic goals, whereas those with high moral identity symbolization are more inclined to pursue extrinsic goals. Thus, intrinsic goal advertising enhances the positive impact of moral identity internalization on consumers’ green purchasing intentions; correspondingly, extrinsic goal advertising enhances the positive effect of moral identity symbolization. The realization of this corollary depends on the extent to which consumers perceive green consumption as a moral event (Feinberg et al., 2019). Therefore, moral awareness enhances the combined effect of moral identity and advertising goal framing on green consumption. Self-consistency emphasizes the fit between consumers’ self-concept and brand/product image, whereas social approval suggests that green consumption can help consumers enhance their image. Self-consistency mediates the combined influence of moral identity internalization and intrinsic goal advertising on green purchase intentions, just as social approval mediates the combined influence of moral identity symbolization and extrinsic goal advertising on green purchase intentions. The aforementioned conclusions are theoretical inferences that have not yet been supported by experimental research.

Based on moral identity theory and SDT, this study combines moral identity, moral awareness, and green advertising goal framing in one research model. This study makes the following theoretical contributions. First, this study found that both moral identity internalization and symbolization can predict green purchasing intention. Second, this study found that moral awareness enhances the combined effect of moral identity internalization and intrinsic goal advertising on green purchasing intention. This finding clarifies the boundary conditions under which moral identity and advertising goal framing jointly influence green purchasing intentions. Finally, this study identified a mediating mechanism by which moral identity and advertising goal framing jointly influence green purchasing intention. This study not only enriches and improves the literature on the formation mechanisms behind consumers’ green purchasing intention but also has substantial practical value for businesses to conduct more effective green marketing by appealing to moral identity and tailoring the framing of green advertising.

The remainder of this paper is organized as follows. First, we briefly survey moral identity theory and define the concept of advertising goal framing. Second, based on existing literature, the hypotheses and framework of this study are presented. Next, we describe the study’s design and methodology, as well as the results of the data analysis. Finally, the conclusions, theoretical contributions, research limitations, and research prospects are discussed.

## Theoretical background and literature review

### Moral identity

The concept of moral identity is derived from social identity and self-concept theories. Aquino and Reed (2002) define moral identity as “a self-concept organized around a set of moral characteristics subordinated to a distinct mental image of how a moral person thinks, feels, and acts.” Based on this concept, moral identity is a trait that also possesses a social reference, which can be a real group of people, a unique perfect, a known person, an obscure individual, or any social structure. Thus, whenever an individual endeavors to view the world in accordance with the hidden meanings of ethical characteristics associated with social structures, they can be assumed to have adopted a moral identity in their social self-schema (Reed, 2002). Aquino and Reed (2002) argued that moral identity should be defined based on moral characteristics, as this allows for differentiation of moral characteristics. Individuals attach different levels of importance to moral characteristics, and their moral identity is only associated with important moral characteristics (Aquino and Reed, 2002). This concept also facilitates the development of instruments to measure moral identity based on moral characteristics (Aquino and Reed, 2002). In conclusion, it demonstrates that a person’s moral identity is not a characteristic of their personality; rather, it can be influenced, either positively or negatively, by contextual, situational, or even personal factors (Aquino and Reed, 2002). An elevated moral identity does not necessarily mean that the possessor is a moral person or holds a high level of morality. Rather, people who have higher levels of internalization of their moral identities are better able to consistently, rapidly, or easily evoke associations with moral traits than those who have lower levels of internalization (Winterich et al., 2013).

Moral identity includes two dimensions: internalization and symbolization. Moral identity internalization reflects a person’s internal moral disposition, while moral identity symbolization represents symbolic expressions of moral character (Aquino and Reed, 2002). Individuals with a high level of internalization of moral identity indulge in ethical behaviors and activities as these align to their subjective moral characteristics, rather than engaging in these behaviors as a means of obtaining public approval (Winterich et al., 2013). Conversely, moral identity symbolization is driven by the need for social approval (Winterich et al., 2013), and is often associated with impression management (Aquino and Reed, 2002). In the literature, the internalization and symbolization of moral identity are not directly compared, and the so-called “high” and “low” dimensions are only compared on a single dimension. Moral identity can be measured using scales or manipulated using writing tasks. For instance, Aquino and Reed (2002) developed 10 items to measure the internalization and symbolization of moral identity. Reed et al. (2007) used a writing task to prime moral identity in their study.

Moral identity has a significant impact on consumer behavior, because moral cognition, moral perceptions, and even feelings of goodwill do not necessarily lead to moral behavior in the absence of direct impact on self-concept (Aquino and Reed, 2002). Moral identity predicts most prosocial behaviors (Patrick et al., 2018) since consumers with a high moral identity are obligated to comply with moral norms associated with their moral self-schema, driven by a fundamental desire for self-consistency (Aquino et al., 2009). For example, moral identity positively influences consumers’ perceptions of corporate charitable donations (Reed et al., 2007). Owing to guilt, consumers with a strong moral identity are more likely to establish associations with ethical brands (Newman and Trump, 2017). Moral identity increases consumers’ propensity to choose green products or strive for green consumption (Wu and Yang, 2018).

### Advertising goal framing

Framing refers to the representation of logically equivalent options in semantically different ways (Krishnamurthy et al., 2001). Depending on the content of the framing, the effects of the content, and how these effects are measured, message framing can be divided into three types: risk selection, attributes, and goal framing. Goal framing is defined as “the behavior or the goal of the behavior is capable of producing a framing effect” (Levin et al., 2002). Goal content theory in SDT distinguishes between consumers’ intrinsic and extrinsic life goals. The achievement of intrinsic life goals triggers personal satisfaction, pleasure, or meaning and can include personal growth, intimacy, feelings of belonging and community, and good health (Kasser and Ryan, 1996). Conversely, extrinsic life goals are related to prioritization of other people’s perceptions, and can include financial success and good reputation and public image (Kasser and Ryan, 1996). This study divided green advertising goal framing into these two categories.

In SDT, intrinsic goals imply autonomous motivation, while extrinsic goals imply controlled motivation. Autonomous motivation implies pleasure and self-harmony, and controlled motivation often implies pain, anxiety, and sadness. Thus, setting intrinsic goals is more effective in achieving learning outcomes than setting extrinsic goals (Vansteenkiste et al., 2004). In consumer behavior research, the advantages of internal goals have clear boundary conditions. For example, consumers who value extrinsic goals over intrinsic ones are more likely to prefer luxury brands (Truong et al., 2010) and use brand names as part of their self-concept (Razmus et al., 2017).

## Research hypothesis and model

### Moral identity, advertising goal framing, and green purchasing intention

Wu and Yang (2018) found that moral identity can trigger a stronger sense of responsibility for environmental damage among consumers, which, in turn, promotes green purchasing intentions. As moral identity internalization and symbolization are dimensions of moral identity, there is also an interactive relationship between these variables and green purchasing intention. Moral identity symbolization encourages prosocial behavior when moral identity internalization is high but not when it is low (Winterich et al., 2013). Consumers with low moral identity internalization and high moral identity symbolization care more if their prosocial behavior is acknowledged (Winterich et al., 2013). However, in contrast, Gotowiec and Mastrigt (2018) found that moral identity symbolization, rather than internalization, predicted all prosocial behaviors. Legere and Kang (2020) studied slow fashion clothing consumption and found that moral identity symbolization had a positive impact on self-promotion and green consumption behavior intention. In a study on social marketing advertising, Lee and Pounders (2019) found that intrinsic goals lead to more positive consumer responses than extrinsic goals, but only for consumers with independent self-construal. This suggests that advertising goal framing and personal traits can jointly influence consumer behavior.

In summary, in their roles as specific dimensions of moral identity, moral identity internalization and symbolization have significant predictive effects on green purchasing intention. However, the manner in which each works is different. For example, consumers with a high level of moral identity symbolization care more about whether their green behavior is publicly recognized than consumers with a high level of moral identity internalization. Therefore, consumers with high moral identity internalization respond more positively to intrinsic goal advertising, whereas those with high moral identity symbolization respond more positively to extrinsic goal advertising.

Based on the above analysis, we propose the following hypotheses:

*H1*: Moral identity positively influences green purchasing intention via the following mechanisms:

*H1a*: Moral identity internalization positively affects green purchasing intention;

*H1b*: Moral identity symbolization positively affects green purchasing intention.

*H2*: Advertising goal framing moderates the positive effect of moral identity on green purchasing intention via the following mechanisms:

*H2a*: Intrinsic goal advertising enhances the positive impact of moral identity internalization on green purchasing intention;

*H2b*: Extrinsic goal advertising enhances the positive impact of moral identity symbolization on green purchasing intentions.

### The moderating role of moral awareness

Moral awareness occurs when a person determines that a situation contains an element that can be evaluated from an ethical standpoint (Reynolds, 2006). According to moral decision theory, moral awareness is an important step in ethical decision-making. Without moral awareness, the moral decision process may not be initiated, because the cognitive structure of the moral decision model is not activated; further, moral awareness ultimately determines whether the behavior is moral or not (Jones, 1991). The greater an individual’s awareness of the moral significance of a situation, the more diagnostic and ethically relevant their knowledge becomes (Aquino et al., 2009).

It has been shown that moral awareness affects consumers’ ethical behavior. Higher moral awareness leads to a stronger negative relationship between moral identity and retaliatory negative word-of-mouth (He and Harris, 2014). Individuals with higher moral awareness will make more negative moral judgments about purchasing counterfeit clothing and thus reduce their purchasing accordingly (Martinez and Jaeger, 2016). The more consumers perceive green consumption as a moral issue, the more likely they are to rely on their moral identity for decision-making, resulting in a stronger relationship between moral identity, advertising goal framing, and green purchasing intention.

Based on the above analysis, we propose the following hypothesis:

*H3*: Moral awareness moderates the combined influence of moral identity and advertising goal framing on consumers' green purchasing intention via the following mechanisms:

*H3a*: Moral awareness enhances the combined influence of moral identity internalization and intrinsic goal advertising on consumers’ green purchasing intentions;

*H3b*: Moral awareness enhances the combined influence of moral identity symbolization and extrinsic goal advertising on consumers’ green purchasing intentions.

### The mediating role of self-consistency and social approval

A key influence on consumer behavior is the development of self-identity (Villarino and Font, 2015). An individual’s thoughts and emotions about themselves as an object constitute their self-concept (Sirgy, 1985). The real, ideal, and social selves are all parts of one’s self-concept (Abimbola et al., 2013). There is evidence that the image of a product user interacts with a consumer’s self-concept to create a subjective experience of self-image/product image, congruence of the self-concept with reality, or self-congruence (Sirgy et al., 1997). Consumers evaluate brands based on how well the symbolic attributes of a brand match their self-concept. Self-consistency can positively influence consumer brand loyalty through sponsorship events and brand sentiment based on corporate sponsorship (Mazodier and Merunka, 2012).

Self-consistency also promotes consumers’ green purchasing intention. For example, Moons et al. (2020) found that self-consistency promotes consumers’ willingness to pay a premium for ecotourism. Self-consistency can also enhance the impact of green self-identity on the perceived value of a product, thereby enhancing consumer acceptance of bioplastics (Confente et al., 2020). In the context of green social enterprise failure, private apologies can lead to higher satisfaction among consumers with higher moral identity internalization, with self-consistency playing a mediating role (Gils and Horton, 2018).

Green brand value incorporates an organization’s economic, social, hedonic, and altruistic values, with social value defined as the perceived utility derived from an association with one or more specific social groups (Papista and Krystallis, 2013). In addition to functional and emotional values, social values promote consumers’ ethical consumption (Kushwah et al., 2019). When a green social enterprise’s service fails to meet expectations, customers with strong moral identity symbolization may be more satisfied with a public apology, and social approval can act as a mediating factor (Gils and Horton, 2018).

Based on the above study, we posit that moral identity internalization drives individuals to strive for green consumption for self-consistent purposes, while moral identity symbolization enhances consumers’ propensity to make environmentally friendly purchases to gain social approval.

Based on the above analysis, we propose the following hypotheses:

*H*4: Self-consistency mediates the combined influence of moral identity internalization and intrinsic goal advertising on green purchasing intention;

*H*5: Social approval mediates the combined influence of moral identity symbolization and extrinsic goal advertising on green purchasing intention.

## Empirical study and data analysis

### Study 1: The main effect of moral identity, advertising goal framing, and the moderating effect of moral awareness

**Figure 1:**
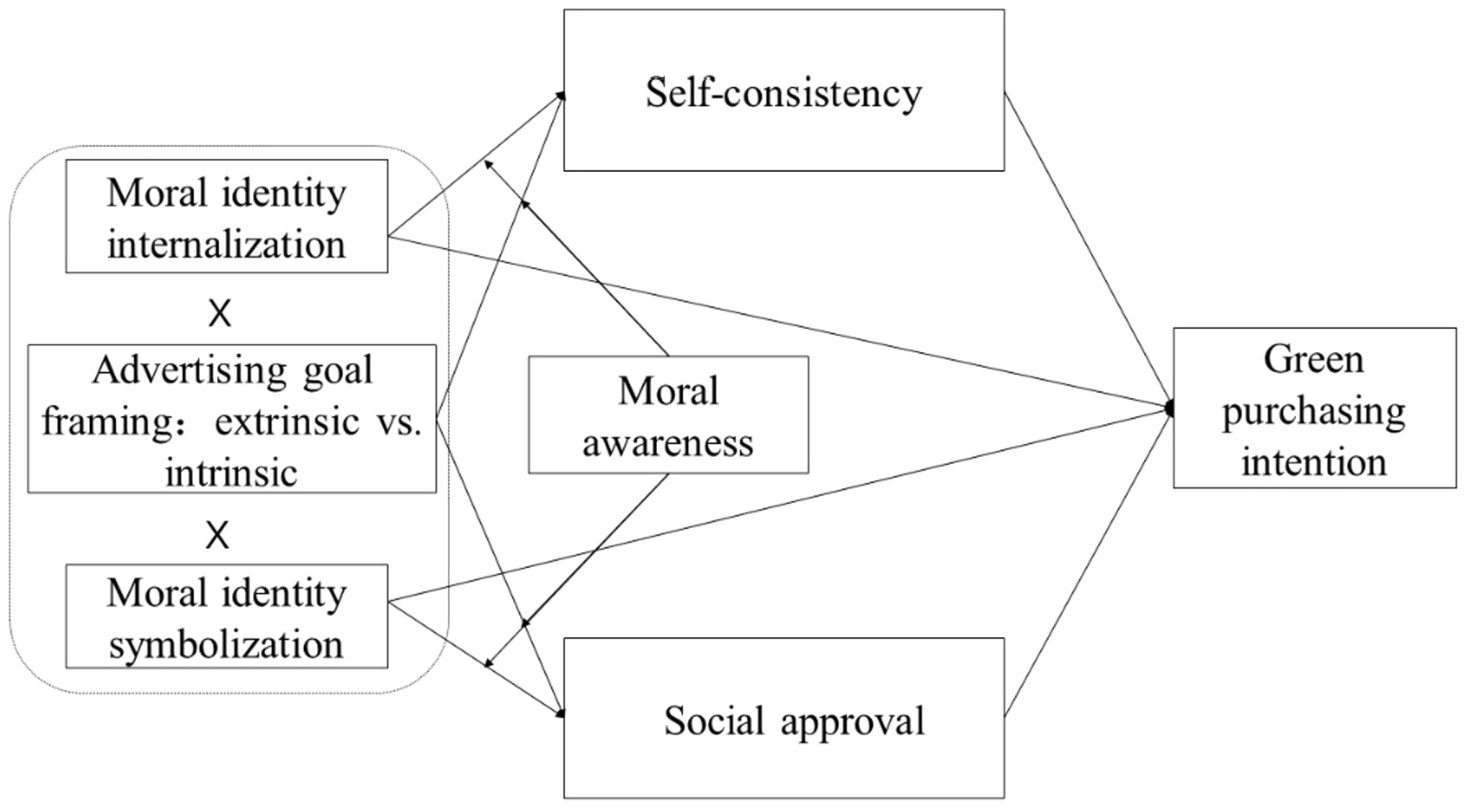
Research model.

The goal of Study 1 was to verify the influence of moral identity and advertising goal framing on consumers’ green purchasing intentions and examine the moderating role of moral awareness. The research model is shown in Figure 1.

#### Experimental design and procedure

The study employed a 2 (moral identity: internalization vs. symbolization) × 2 (advertising goal framing: intrinsic vs. extrinsic) × 2 (moral awareness: low vs. high) between-group design. The experiment first assessed the participants’ internalization and symbolization of moral identity. To avoid the influence of actual brands on consumers, this study featured a nonexistent clothing brand, LM, which introduced an environmentally conscious clothing line made from materials such as natural cotton, natural cloth, Tencel, and polyester. Advertising for this environmentally conscious clothing line was created with consistent content, excepting differing goal framing. Participants in the intrinsic goal group were presented with the slogan: “Wearing nature on your body makes you healthier and more comfortable.” Those in the extrinsic goal group read the advertising slogan: “Wearing nature on your body makes you more fashionable and attractive.” We then measured the participants’ green purchasing intentions, moral awareness, and demographic information.

The moral identity scale was based on the findings of Aquino and Reed (2002). First, the questionnaire presented the participants with morally relevant words such as “considerate,” “compassionate,” “fair,” and “friendly.” We then measured participants’ degree of internalization *via* six items, such as “It would make me feel good to be a person who has these characteristics” and “Being someone who has these characteristics is an important part of who I am” (Cronbach’s *α* = 0.765). We assessed participants’ moral identity symbolization *via* six items, such as “I often buy products that communicate the fact that I have these characteristics” and “I often wear clothes that identify me as having these characteristics” (Cronbach’s *α* = 0.754).

The green purchasing intention scale used in this study was adapted from Wang et al.'s (2017) study and included four items, such as “I would collect and learn more about approximately this eco-friendly clothing” and “I would recommend this eco-friendly clothing to a companion” (Cronbach’s *α* = 0.733).

The moral awareness scale used in this study was adapted from Reynolds (2006) and included three items: “Green consumption decisions involve moral considerations,” Green consumption involves ethical or moral issues,” and “Green consumption can be described as an ethical issue” (Cronbach’s *α* = 0.753). Participants’ level of agreement with these items was assessed using a 7-point Likert scale. Table 1 presents the scales and items used in study 1.

**Table 1:** Scales and Items used in study 1.

Scales and Items used in study 1
Moral identity internalization (1 = strongly disagree; 7 = strongly agree; Cronbach’s *α* = 0.765).
It would make me feel good to be a person who has these characteristics.
Being someone who has these characteristics is an important part of who I am.
I would be ashamed to be a person who had these characteristics (*R*).
Having these characteristics is not really important to me (*R*).
I strongly desire to have these characteristics.
Moral identity symbolization (1 = strongly disagree; 7 = strongly agree; Cronbach’s *α* = 0.754).
I often buy products that communicate the fact that I have these characteristics.
I often wear clothes that identify me as having these characteristics.
The types of things I do in my spare time (e.g., hobbies) clearly identify me as having these characteristics.
The kinds of books and magazines that I read identify me as having these characteristics.
The fact that I have these characteristics is communicated to others by my membership in certain organizations.
I am actively involved in activities that communicate to others that I have these characteristics.
Green purchasing intention(1 = strongly disagree; 7 = strongly agree; Cronbach’s *α* = 0.733)
I would collect and learn more approximately this eco-friendly clothing.
I would suggest this eco-friendly clothing to a companion.
I will consider buying the green clothing when I need it.
The ad will prompt me to buy the green clothing.
Moral awareness (1 = strongly disagree; 7 = strongly agree; Cronbach’s *α* = 0.753).
Green consumption decisions involve moral considerations.
Green consumption involves ethical or moral issues.
Green consumption can be described as an ethical issue.

We generated the questionnaire including the aforementioned scales using WJX and provided participants with a link. The questionnaires were collected over the course of 1 week, and participants who did not respond fully or failed the screening questions were excluded. A total of 267 valid questionnaires were obtained, including 172 females (64.4%) aged between 16 and 57 years.

#### Data analysis and conclusion

The study used SPSS (version 21.0) for statistical analysis of the data. One-way analysis of variance (ANOVA) revealed that participants in the intrinsic and extrinsic goal groups did not differ significantly by gender (*M*_intrinsic_ = 1.65, SD = 0.478, *M*_extrinsic_ = 1.64, SD = 0.483, *F*(1,265) = 0.061, *p* = 0.806) and age (*M*_intrinsic_ = 28.90, SD = 7.082, *M*_extrinsic_ = 28.88, SD = 7.403, *F*(1,265) = 0.001, *p* = 0.970). The study excluded the effects of sex and age.

First, we converted goal framing into a dummy variable (intrinsic goal = 0; extrinsic goal = 1), mean-centralized moral identity internalization, symbolization, and moral awareness, and constructed interaction terms between the three variables. Using green purchasing intention as the dependent variable; advertising goal framing, moral identity internalization, moral awareness, and their interaction terms as independent variables; and moral identity symbolization as control variables, hierarchical regression analysis was conducted. The regression results show that the internalization of moral identity had a critical positive impact on green purchasing intention (*β* = 0.593, *t* = 7.970, *p* < 0.001). The interactive impact of moral identity internalization and advertising goal framing on green purchasing intention was significant (*β* = −0.254, *t* = −3.514, *p* < 0.01). The interactive effect of internalization of moral identity, advertising goal framing, and moral awareness on green purchasing intention was significant (*β* = −0.285, *t* = −3.388, *p* < 0.01). Hypotheses H1a, H2a, and H3a were verified. The results of the regression analysis are presented in Table 2.

**Table 2:** Analysis of the moderating role of moral awareness.

Variable		Model 1		Model 2		Model 3	
		*β*	*t*	*β*	*t*	*β*	*t*
Control variable	Moral identity symbolization	0.493^***^	9.670	0.481^***^	9.484	0.480^***^	9.651
Independent variable	Moral identity internalization	0.384^***^	7.739	0.517^***^	6.594	0.593^***^	7.970
Moderating variable	Advertising goal framing	−0.111	−2.739	−0.112^**^	−2.813	−0.063	−1.500
	Moral awareness	−0.007	−0.139	−0.158^*^	−2.214	−0.186^**^	−2.645
Moral identity internalization*advertising goal framing				−0.155^*^	−2.295	−0.254^**^	−3.514
Moral identity internalization*advertising goal framing*moral awareness						−0.285^**^	−3.388
	*F*-value	89.746^***^		54.098^***^		50.723^***^	
Regression model	*R* ^2^	0.583		0.599		0.616	
Abstract	Δ*F*-value	89.746^***^		3.322^*^		11.479^**^	
	Δ*R*^2^	0.583		0.016		0.017	

* *p* < 0.05,

** *p* < 0.01, and

*** *p* < 0.001.

*N* = 267; advertising goal framing is a dummy variable; 0 = intrinsic goal, 1 = extrinsic goal.

Second, we constructed interaction terms between advertising goal framing, moral identity symbolization, and moral awareness. Hierarchical regression was conducted, with green purchasing intention as the dependent variable; advertising goal framing, moral identity symbolization, moral awareness, and interaction terms as the independent variables; and internalization of moral identity as the control variable. The regression results indicate that moral identity symbolization has a significant positive effect on green purchasing intention (*β* = 0.513, *t* = 7.724, *p* < 0.001). The interactive effect of moral identity symbolization and advertising goal framing on green purchasing intention was insignificant (*β* = −0.032, *t* = −0.487, *p* = 0.627). The interactive effects of moral identity symbolization, advertising goal framing, and moral awareness on green purchasing intention were insignificant (*β* = −0.040, *t* = −0.560, *p* = 0.576). Hypothesis H1b was confirmed, while hypotheses H2b and H3b were not, indicating that the level of moral identity symbolization of consumers is unaffected by advertising goal framing.

### Study 2: The mediating role of self-consistency and social approval

Experiment 2 verified that self-consistency and social approval play a mediating role in the combined influence of moral identity and advertising goal framing on consumers’ green purchasing intentions.

#### Experimental design and procedure

This study employed a 2 (moral identity: internalization vs. symbolization) × 2 (advertising goal framing: intrinsic vs. extrinsic) between-group design. First, we assessed participants’ internalization and symbolization of moral identity. The study featured a nonexistent food company, LK, which produces organic milk.

Second, a questionnaire was used to introduce organic milk to the participants. Organic milk was advertised as “natural” and “pollution-free,” and the use of chemical fertilizers, pesticides, hormones, development control agents, fortification additives, nutritional additives, and other chemical products is eliminated from its manufacture. Packaging, storage, and transportation are strictly compliant with the relevant standards for organic food. The advertising of this brand of organic milk was employed as the experimental stimulus material. The advertisements included pictures and jingles and were consistent between the experimental groups, except differences in goal framing. Participants in the intrinsic goal group were presented with the slogan: “The gift of nature gives you energy and a healthy body.” Those in the extrinsic goal group were presented with the slogan: “The gift of nature gives you glow and radiance.” We then measured participants’ self-consistency, social approval, green purchasing intention, and demographic information.

Aquino and Reed’s moral identity scale (Aquino and Reed, 2002), which includes 12 measurement items, was used to assess moral identity internalization (Cronbach’s *α* = 0.761) and moral identity symbolization (Cronbach’s *α* = 0.758).

The self-consistency scale was derived from Mazodier and Merunka (2012) and consists of three items: “I feel I am part of a family that consumes organic milk,” “People who buy organic milk are similar to me,” and “Buying organic milk reflects who I am as a person” (Cronbach’s *α* = 0.723).

The social approval assessment instrument used in this study was adapted from Kushwah et al.'s (2019) study and included four items such as: “Buying organic milk helps me gain social approval” and “Buying organic milk makes a good impression on others” (Cronbach’s *α* = 0.865).

Participants’ green purchasing intention was assessed using a tool adapted from Wang et al. (2017), which included four measurement items (Cronbach’s *α* = 0.720). Table 3 presents the scales and items used in study 2.

**Table 3:** Scales and Items used in study 2.

Scales and Items used in study 2
Moral identity internalization (1 = strongly disagree; 7 = strongly agree; Cronbach’s *α* = 0.761)
Same scales and items as in study 1.
Moral identity Symbolization(1 = strongly disagree; 7 = strongly agree; Cronbach’s *α* = 0.758).
Same scales and items as in study 1.
Self-consistency (1 = strongly disagree; 7 = strongly agree; Cronbach’s *α* = 0.723)
I feel I am part of a family that consumes organic milk.
People who buy organic milk are similar to me.
Buying organic milk reflects who I am as a person.
Social approval (1 = strongly disagree; 7 = strongly agree; Cronbach’s *α* = 0.865).
Buying organic milk helps me gain social approval.
Buying organic milk makes a good impression on others.
Buying organic milk affects how people perceive me.
Buying organic milk makes me feel accepted by others.
Green purchasing intention (1 = strongly disagree; 7 = strongly agree; Cronbach’s *α* = 0.720).
I will collect and learn more about this organic milk.
I would recommend this organic milk to a friend.
I will consider buying this organic milk when I need it.
The ad will motivate me to buy the organic milk.

As above, questionnaires were generated and distributed using the WJX.cn website. We received 235 valid responses, including 143 from women (54.2%). All participants were aged 18–52 years.

#### Data analysis and conclusion

The study used SPSS (version 21.0) for the statistical analysis of the data. A one-way ANOVA revealed that participants in the intrinsic and extrinsic goal groups did not differ significantly in sex (*M*_intrinsic_ = 1.62, SD = 0.486, *M*_extrinsic_ = 1.59, SD = 0.493, *F*(1,233) = 0.231, *p* = 0.631) and age (*M*_intrinsic_ = 30.83, SD = 8.172, *M*_extrinsic_ = 29.92, SD = 6.301, *F*(1,233) = 0.882, *p* = 0.349). The study excluded the effects of sex and age.

First, the combined influence of moral identity and advertising goal framing on consumers’ green purchasing intentions was verified. Goal framing was converted into a dummy variable (intrinsic goal = 0, extrinsic goal = 1), moral identity was mean-centralized, and the interaction term between advertising goal framing and moral identity was constructed. Hierarchical regression was conducted, with green purchasing intention as the dependent variable; advertising goal framing, internalization of moral identity, and their interaction terms as the independent variables; and moral identity symbolization as the control variable. The regression results showed that internalization of moral identity had a significant positive effect on green purchasing intention (*β* = 0.558, *t* = 8.145, *p* < 0.001), and the interactive effect of internalization of moral identity and advertising goal framing on consumers’ green purchasing intention was significant (*β* = −0.119, *t* = −2.183, *p* < 0.05). Hierarchical regression was again conducted for moral identity symbolization, with green purchasing intention as the dependent variable; advertising goal framing, moral identity symbolization, and its interaction term as independent variables; and moral identity internalization as control variables. The regression results showed that moral identity symbolization had a significant positive effect on green purchasing intention (*β* = 0.426, *t* = 6.613, *p* < 0.001), while the interactive effect of moral identity symbolization and advertising goal framing on green purchasing intention was insignificant (*β* = −0.022, *t* = −0.378, *p* = 0.706). The results of data analysis validated the findings of Experiment 1.

Second, the mediating role of self-consistency and social approval was verified. The PROCESS plug-in was used to analyze the mediating effects. Following Chen et al. (2013), we chose Model 8 and set the bootstrapping sample size to 5,000, with consumer green purchasing intention as the dependent variable; moral identity internalization as the independent variable; advertising goal framing as the moderating variable; self-consistency as the mediating variable; and moral identity symbolization as the control variable. Data analysis results showed that the 95% confidence intervals of self-consistency for both intrinsic (LLCI = 0.0330, ULCI = 0.1684) and extrinsic goals (LLCI = 0.0179, ULCI = 0.1367) did not include 0, indicating that self-consistency mediates the combined effect of internalization of moral identity and advertising goal framing on green purchasing intention. We repeated the same process for model identity symbolization, with consumers’ green purchasing intention as the dependent variable; moral identity symbolization as the independent variable; advertising goal framing as the moderating variable; social approval as the mediating variable; and internalization of moral identity as the control variable. The 95% confidence intervals for both the intrinsic goal (LLCI = 0.0152, ULCI = 0.2880) and extrinsic goal (LLCI = 0.0159, ULCI = 0.2882) excluded 0, suggesting a mediating role of social approval in the positive effect of moral identity symbolization and advertising goal framing on green purchasing intention. Hypotheses H4 and H5 were verified. Table 4 presents the results of the mediating effect analysis.

**Table 4:** Analysis of the mediating role of self-consistency and social approval.

Mediating variable	Advertising goal framing	Effect	Boot SE	Boot LLCI	Boot ULCI
self-consistency	Intrinsic goal	0.0903	0.0337	0.0330	0.1684
	Extrinsic goal	0.0642	0.0290	0.0179	0.1367
Social approval	Intrinsic goal	0.1495	0.0684	0.0152	0.2880
	Extrinsic goal	0.1461	0.0683	0.0159	0.2882

*N* = 235; Bootstrap = 5,000.

## Discussion

Experiment 1 verified the positive effect of moral identity on consumers’ green purchasing intentions and verified that consumers with a high degree of moral identity internalization respond more positively to advertising appealing to intrinsic goals. Moral awareness improves the combined effect of moral identity internalization and advertising goal framing on consumers’ green purchasing intentions. Experiment 2 confirmed the combined influence of moral identity internalization and intrinsic goal advertising on green purchasing intentions, and the role of self-consistency and social approval as mediators. Table 5 presents the results of hypothesis validation.

**Table 5:** Results of hypothesis testing.

Hypotheses	Path coefficient and *p*-value	Decision
*H*1a: Moral identity internalization positively affects green purchasing intention	*β* = 0.593; *p* < 0.001	supported
*H*1b: Moral identity symbolization positively affects green purchasing intention	*β* = 0.513; *p* < 0.001	supported
*H*2a: Intrinsic goal advertising enhances the positive impact of internalization of moral identity on green purchasing intention	*β* = −0.254; *p* < 0.01	supported
*H*2b: Extrinsic goal advertising enhances the positive impact of moral identity symbolization on green purchasing intention	*β* = −0.032; *p* = 0.627	rejected
*H*3a: Moral awareness enhances the combined influence of moral identity internalization and intrinsic goal advertising on consumers’ green purchasing intention	*β* = −0.285; *p* < 0.01	supported
*H*3b: Moral awareness enhances the combined influence of moral identity symbolization and extrinsic goal advertising on consumers’ green purchasing intention	*β* = −0.040; *p* = 0.576	rejected
*H*4: Self-consistency mediates the combined influence of moral identity internalization and intrinsic goal advertising on green purchasing intention		supported
*H*5: Social approval mediates the combined influence of moral identity symbolization, extrinsic goal advertising on green purchasing intention		supported

### Conclusion

First, moral identity can promote consumers’ green purchasing intentions. The current experiments showed that the internalization and symbolization, two dimensions of moral identity, could promote consumers’ green purchasing intentions. It was also found that moral identity internalization and advertising goal framing jointly influenced green purchasing intentions. This means that intrinsic goal advertising enhances the positive effect of moral identity internalization on consumers’ green purchasing intentions, compared to extrinsic goal advertising.

Second, moral awareness enhances the combined influence of moral identity internalization and intrinsic goal advertising on consumers’ green purchasing intentions. This suggests that the more consumers are aware that green consumption is ethical, the more they will strive for it, driven by their moral identity and intrinsic motivation.

Finally, self-consistency mediates the positive effect of moral identity internalization on consumers’ green purchasing intentions for both intrinsic and extrinsic green advertising goals. Social approval mediates the positive effect of moral identity symbolization on consumers’ green purchasing intentions for both intrinsic and extrinsic green advertising goals.

### Research contribution

#### Theoretical contribution

First, the literature has focused on the role of internalization of moral identity in promoting green consumption, ignoring the symbolization of moral identity. Existing studies have shown that the internalization of moral identity can promote green consumption (Wu and Yang, 2018). However, whether the symbolization of moral identity has the same effect as the internalization of moral identity has not yet been verified. This study considers the role of internalization and symbolization of moral identity and finds that both can promote green purchasing intentions.

Second, in contrast to existing studies, this study not only focuses on the role of moral identity but also assesses the impact of advertising goal framing, to examine the combined role of personality traits and external stimuli in green consumption. Existing studies have demonstrated the positive effects of goal framing on green consumption. For example, Yang et al. (2015) classified green consumption goal framing into gain, hedonic, and normative goals. Unlike Yang et al. (2015), we focused on advertising goal framing. Based on goal content theory, this study classifies advertising goal framing into intrinsic and extrinsic goals. The combined effect of moral identity and advertising goal framing on green purchasing intention was identified and demonstrated through experimental methods and statistical analysis. This study found that consumers with a highly internalized moral identity had more positive responses to intrinsic goal advertising.

Third, existing research has identified a positive effect of moralization on green consumption (Feinberg et al., 2019), but this study explores green curtailment behavior, rather than purchase behavior, and does not consider the combined effect of moralization with other variables. The current findings suggest that moral awareness reflects consumers’ level of moralization regarding green consumption. It was identified that moral consciousness reinforces the combined effect of internalized moral identity and intrinsic goal advertising on green purchasing intentions.

Lastly, Wu and Yang (2018) found a mediating role of perceived responsibility for environmental damage in the positive effect of moral identity on green purchasing intentions. Unfortunately, this study only considered moral identity internalization. Regarding the concepts of moral identity internalization and symbolization, it is not difficult to infer that the mechanisms behind their respective roles in promoting green consumption differ. This study found that self-consistency plays a mediating role in the combined effect of moral identity internalization and intrinsic goal advertising on green purchasing intentions. There is a mediating role of social approval in the combined effect of moral identity symbolization and extrinsic goal advertising on green consumption.

#### Implications

First, companies should tailor their moral identity to promote consumers’ green purchasing intentions when engaging in green marketing. To strengthen the positive role of moral identity, green advertising should emphasize how green consumption can help consumers to achieve intrinsic goals.

Second, companies must encourage consumers to view green consumption as a moral behavior, to increase the effectiveness of moral identity and intrinsic goal advertising. When consumers incorporate green consumption into their ethical decision-making processes, the internalization of moral identity and the appeal of advertising to intrinsic goals will promote consumers’ green consumption tendencies.

Finally, companies can emphasize the social value of green consumption appropriately. Many green products, such as environmentally friendly clothing and vehicles, are visible to a consumer’s peers, and green marketing for these products can motivate consumers to gain social approval by purchasing these items.

### Research prospects

This study has some limitations to be addressed. This study mainly used experimental and questionnaire methods to verify the research hypotheses, and more diversified methods, such as field experiments and panel data analysis, should be used in the future. Additionally, the stimulus materials used included only clothing and food, and a broader range of product categories should be incorporated in the future.

## Data availability statement

The original contributions presented in the study are included in the article/supplementary material, further inquiries can be directed to the corresponding author.

## Author contributions

DL: conceptualization, methodology, software, formal analysis, investigation, resources, data curation, writing—original draft preparation, and funding acquisition. DL, GC, and CW: validation. GC and CW: writing—review and editing, supervision, and project administration. All authors contributed to the article and approved the submitted version.

## Funding

This research was funded by the National Social Science Foundation of China (grant no. 18AGL010), Social Science Foundation of the Education Department of Henan Province (grant no. 2023-ZZJH-019), and Social Science Foundation of Henan Polytechnic University (grant no. SKB2022-07).

## Conflict of interest

The authors declare that the research was conducted in the absence of any commercial or financial relationships that could be construed as a potential conflict of interest.

## Publisher’s note

All claims expressed in this article are solely those of the authors and do not necessarily represent those of their affiliated organizations, or those of the publisher, the editors and the reviewers. Any product that may be evaluated in this article, or claim that may be made by its manufacturer, is not guaranteed or endorsed by the publisher.
